# Inferior vena cava filter use at a large community hospital: a retrospective cohort study

**DOI:** 10.1038/s41598-024-60868-z

**Published:** 2024-05-03

**Authors:** Stephanie Fontyn, Yuxin Bai, Samantha Bolger, Kaity Greco, Tzu-Fei Wang, Caroline Hamm, Andrea Cervi

**Affiliations:** 1 Schulich School of Medicine and Dentistry, London, ON Canada; 2 Department of Medicine, University of Ottawa at The Ottawa Hospital and Ottawa Hospital Research Institute, Ottawa, Canada; 3 Department of Medical Oncology, Windsor Regional Cancer Centre, 1995 Lens Avenue, Windsor, ON N8W 1L9 Canada

**Keywords:** Haematological diseases, Health care, Therapeutics

## Abstract

Inferior vena cava (IVC) filters are considered when patients with venous thromboembolism (VTE) develop a contraindication to anticoagulation. Use of IVC filters is increasing, despite associated complications and lack of data on efficacy in reducing VTE-related mortality. We characterized the pattern of IVC filter use at a large community hospital between 2018 and 2022. Specifically, we assessed the indications for IVC filter insertion, filter removal rates, and filter-associated complications. Indications for IVC filters were compared to those outlined by current clinical practice guidelines. We reviewed 120 consecutive filter placement events. The most common indications included recent VTE and active bleeding (40.0%) or need for anticoagulation interruption for surgery (25.8%). Approximately one-third (30.0%) of IVC filters were inserted for indications either not supported or addressed by guidelines. Half (50.0%) of patients had successful removal of their IVC filter. At least 13 patients (10.8%) experienced a filter-related complication. In a large community-based practice, nearly one-third of IVC filters were inserted for indications not universally supported by current practice guidelines. Moreover, most IVC filters were not removed, raising the risk of filter-associated complications, and supporting the need for development of comprehensive guidelines addressing use of IVC filters, and post-insertion monitoring practices.

## Introduction

Anticoagulation remains the first-line treatment for acute deep vein thrombosis (DVT) and pulmonary embolism (PE)^1^; although, use of inferior vena cava (IVC) filters has risen over recent decades^2^. IVC filters were originally designed to trap thrombus originating from lower extremity veins and prevent the development of clinically-significant PE. Contraindications to anticoagulation, such as major bleeding or need for surgical intervention among recently diagnosed venous thromboembolism (VTE), remain the most consistent indications for IVC filter placement^3–6^. Additional scenarios where filters are considered in clinical practice are neither addressed nor universally supported by professional bodies’ guidelines.

The majority of evidence informing on IVC filter use comes from retrospective reviews or case series^7^. Two major randomized controlled trials (RCTs) evaluating use of filters, A Clinical Trial of Vena Caval Filters in the Prevention of Pulmonary Embolism in Patients with Proximal Deep-Vein Thrombosis (PREPIC) and Effect of a Retrievable Inferior Vena Cava Filter Plus Anticoagulation versus Anticoagulation Alone on Risk of Recurrent Pulmonary Embolism: A Randomized Clinical Trial (PREPIC2), failed to demonstrate a benefit of IVC filters among individuals receiving anticoagulation, although risk of recurrent DVT was increased among patients who received a filter^8,9^. Furthermore, RCTs evaluating the prophylactic placement of filters in trauma patients with a contraindication to anticoagulation prophylaxis failed to demonstrate a reduction in symptomatic PE^10,11^. However, there have been no RCTs assessing the use of IVC filters in patients with VTE who have a contraindication to receiving therapeutic anticoagulation.

With continued improvements in IVC filter technology, the use of IVC filters has risen in recent decades despite a lack of data on the effectiveness of filters in reducing VTE-related mortality^2,12,13^. Moreover, IVC filters are associated with significant morbidity, including filter migration, recurrent lower extremity DVT, IVC thrombosis, and fatal bleeding^8,14–16^. Once patients have safely resumed anticoagulation, IVC filters should be evaluated for removal to minimize the risk of complications^2,17^. Most complications from filters occur following 30 days of insertion, and prolonged insertion increases the likelihood of failed filter retrieval and chronic morbidity^14,18^. A systematic review of IVC filter use found that the majority of IVC filters are never removed, with rates of removal ranging from 12 to 42%^14^. Finally, the financial burden of IVC filters must be considered, as previous studies have shown that filters are associated with a net financial loss under various reimbursement strategies^19,20^.

This study aimed to understand the pattern of IVC filter use among patients with venous thrombotic disease at Windsor Regional Hospital, a large community-based hospital with academic affiliations in Ontario, Canada. Specifically, the indications for IVC filter insertion, rates of IVC filter removal, and filter-associated complications were characterized. Indications for filter insertion were compared to those outlined by major clinical practice guidelines.

## Methods

We conducted a retrospective chart review of adult patients (aged 18 years and older) who had an IVC filter inserted between August 1, 2018 and February 15, 2022 at Windsor Regional Hospital (WRH). This timeframe was chosen to allow sufficient time for patient follow-up and capture filter-related outcomes. Patients were identified using ICD10 codes for the insertion of IVC filters. This study was granted Category A approval by the Windsor Regional Hospital Research Ethics Board, and all methods were carried out with relevant guidelines and regulations. The requirement for informed consent from the study subjects were waived by the institutional review board of WRH Research Ethics Board due to the retrospective study design.

REDCap® software (version 12.5.4) was used to collect and store patient data from WRH electronic health records. The last day of follow-up corresponded to either the IVC filter removal date or, in patients who did not have their IVC filter removed, the last date of documentation found in the electronic medical records. For patients who died while their filter was in-situ, the date of death was noted instead.

Indications for IVC filter insertion at WRH were compared to those outlined by the 2021 updated 9th edition of ACCP (American College of Chest Physicians), 2011 edition of AHA (American Heart Association) and 2020 edition of SIR (Society of Interventional Radiology)^3–6^. These three guidelines were chosen by consensus as the most comprehensive available and reflective of expert opinion across a variety of specialties including Thrombosis, Vascular Medicine, Interventional Radiology, Cardiology, etc.

Patient demographics, anticoagulation use, filter removal rates, and filter outcomes were summarized descriptively using median, interquartile range (IQR), and frequency. Chi-square tests of independence were performed to examine the relationship between filter-related complications and the appropriateness of filter insertion, presence of active cancer and duration until removal.

### Ethics approval

Category A approval was granted by the Windsor Regional Hospital Research Ethics Board. (January 26, 2022 /REB #22-422).

## Results

### Characteristics of patients who received an inferior vena cava filter

We reviewed 120 consecutive IVC filter placement events in 118 patients with a median duration of follow-up of 53 (IQR: 20–170) days. All IVC filters in this review were retrievable Cook Celect filters. Two patients received an IVC filter during two different occurrences (Table 1). Seventy-six (64.4%) patients were male and forty-two (35.6%) female, with a median age of 68 years (IQR: 58–80) at the time of filter placement. The majority of patients had at least one VTE risk factor (n = 90; 75.0%), with the most common risk factor being active malignancy in 45.0% of patients (n = 54). Additional risk factors included: history of VTE (n = 34; 28.3%), surgery within 3 months of VTE diagnosis (n = 27; 22.5%), estrogen use (n = 1; 0.83%), and inherited thrombophilia (n = 1; 0.83%).

**Table 1:** Characteristics of patients who received an inferior vena cava filter.

Patient demographic (n = 118)	n (%)
*Sex*	
Male	76 (64.4)
Female	42 (35.6)
Age (years)	67.8 (IQR: 58–80)
Deceased	57 (48.3)
*Cause of death*	
Cancer	21 (17.8)
Unknown	14 (11.9)
Multi-organ failure	8 (6.8)
Cerebrovascular/cardiovascular disease	4 (3.4)
Infection	4 (3.4)
Respiratory failure	4 (3.4)
Post-operative complication	2 (1.7)
Characteristics of Filter Placement Events (n = 120)	n (%)
Type of VTE	n (%)
DVT alone	59 (49.2)
PE alone	38 (31.7)
DVT & PE	23 (19.2)
Presence of VTE Risk Factors	90 (75.0)
Active malignancy	54 (45.0)
History of prior VTE	34 (28.3)
Surgery within 3 months	27 (22.5)
Estrogen use	1 (0.83)
Inherited thrombophilia^a^	1 (0.83)

IQR, interquartile range; VTE, venous thromboembolism; DVT, deep vein thrombosis; PE, pulmonary embolism.

^a^Heterozygous for factor V Leiden gene mutation.

Lower extremity DVT (n = 59; 49.2%) was the most common type of VTE experienced by the patients in our study. Approximately one third of patients who received an IVC filter (n = 38; 31.7%) had a PE without concurrent lower extremity DVT, and 19.2% (n = 23) had both DVT and PE (Table 1). All DVT and PE were objectively confirmed by imaging and documented prior to IVC filter placement. Most patients (n = 61; 57.0%) had their VTE diagnosed within 1 week of IVC filter insertion, 13.1% (n = 14) within 2 weeks, 10.3% (n = 11) within 4 weeks, and 18.7% (n = 20) within 3 months of IVC filter insertion.

Fifty-seven (48.3%) patients died during the course of follow-up. The most common cause of death was cancer (n = 21; 17.8%), followed by multi-organ failure (n = 8; 6.8%), cerebrovascular/cardiovascular disease (n = 4; 3.4%), infection (n = 4; 3.4%), respiratory failure (n = 4; 3.4%), and post-operative complications (n = 2; 1.7%). Cause of death was unknown in 14 patients (11.9%); in two of these patients, the IVC filter was removed prior to death after a median of 970 days (IQR: 823–1113). Two patients died due to complications from lower gastrointestinal bleeding. One of the two patients never resumed anticoagulation and therefore had no anticoagulants prior to hemorrhage. The second patient’s anticoagulation was held within 24 h of death.

### Indications for filter insertion at Windsor Regional Hospital

The most common indication for IVC filter insertion in our study was recent VTE, defined as DVT and/or PE diagnosed within 3 months, and active bleeding (n = 48; 40.0%) (Table 2). Active bleeding was a major bleeding event defined according to the International Society on Thrombosis and Haemostasis (ISTH) criteria^21^. Active bleeding events included: intra-abdominal bleed (n = 23; 47.9%), intracranial hemorrhage (n = 12; 25.0%), hematuria (n = 6; 12.5%), intramuscular hematoma (n = 3; 6.3%), hemorrhagic stroke (n = 2; 4.2%), spinal hematoma (n = 1; 2.1%), and hemorrhage post-open fracture (n = 1; 2.1%).

**Table 2:** Comparison of IVC filter indications at Windsor Regional Hospital to clinical practice guidelines (n = 120).

Indication for IVC filter	IVC filter indication at WRH, n (%)	Does the guideline support the indication for IVC filter use?		
		ACCP	SIR	AHA
Recent VTE & major bleeding	48 (40)	Yes	Yes	Yes
Recent VTE & interruption of anticoagulation for surgery	32 (26.7)	Yes	Yes	Yes
Recent VTE & perceived risk of bleeding with no active bleed	11 (9.2)	No	Not addressed	No
Non-acute VTE & active bleeding	10 (8.3)	Not addressed	Not addressed	Not addressed
Non-acute VTE & interruption of anticoagulation for surgery	10 (8.3)	Not addressed	No	Not addressed
Recent VTE & non-major bleed with ongoing anticoagulation	5 (4.2)	Not addressed	Not addressed	Not addressed
Recent VTE & severe thrombocytopenia^a^	2 (1.7)	Yes	Yes	Yes
Anticoagulation failure	1 (0.8)	Not addressed	No	Yes
Massive PE & residual DVT	1 (0. 8)	Not addressed	Not addressed	Yes

ACCP, American College of Chest Physicians; AHA, American Heart Association; SIR, Society of Interventional Radiology.

^a^Platelet count less than 20 × 10^9^/L.

Recent VTE with a need for anticoagulation interruption for major surgery was the second most common indication for IVC filter insertion (n = 32; 26.7%) (Table 2). Thirty percent of IVC filters (n = 36) were inserted for indications that were either not addressed or supported by at least one of the three major guidelines (ACCP, AHA, SIR) (Table 2). These indications included: (i) need for interruption of anticoagulation in patients with a remote history of VTE (VTE greater than 3 months ago) and active bleed (n = 10; 8.3%) or prior to surgery (n = 10; 8.3%), (ii) recent VTE and perceived risk of bleeding with no active bleed or contraindication to anticoagulation (n = 11; 9.2%), and (iii) recent VTE and non-major bleed with ongoing use of anticoagulant therapy (n = 5; 4.2%).

### Anticoagulant management before and after filter placement

The direct oral anticoagulants (DOACs) were the most common type of anticoagulant used before IVC filter insertion (n = 38, 31.7%) (Table 3). Anticoagulant therapy was resumed in 71 patients (59.2%) after a median of 6 days (IQR: 1–13) following IVC filter insertion. Among the 60 patients (50.0%) with a chronic indwelling IVC filter, 22 remained on anticoagulation in the form of a DOAC, vitamin K antagonist (VKA), or low molecular weight heparin (LMWH).

**Table 3:** Anticoagulant therapy use pre- and post-IVC filter insertion.

Anticoagulant type	Pre-IVC filter insertion n (%)	Post-IVC filter insertion n (%)
DOAC	38 (31.7)	41 (34.2)
LMWH	30 (25.0)	28 (23.3)
Unfractionated heparin	9 (7.5)	0 (0.0)
Vitamin K antagonist	7 (5.8)	2 (1.7)
None	36 (30.0)	49 (40.8)

DOAC, direct oral anticoagulant; IVC, inferior vena cava; LMWH, low molecular weight heparin.

### Outcomes of inferior vena cava filters

Half (50.0%, n = 60) of patients had successful removal of their IVC filter with a mean of 1.2 (IQR: 1–1) attempts at filter removal per patient. Six patients (5.0%) did not have their filter removed after 1 or more attempts at removal. Filters were not removed due to presence of thrombus at the filter at the time of removal (n = 3, 2.5%), filter was tilted or embedded in the caval wall and not retrievable (n = 2, 1.7%), or renal function precluded use of intravenous contrast necessary for the procedure (n = 1, 0.8%). IVC filter retrieval was not attempted in 45.0% of patients (n = 54), of which 47 (87.0%) died throughout the course of chart review and 7 (13.0%) were lost to follow-up based on our last date of chart review. Fifty (42.4%) patients died with an IVC filter in place due to reasons not related to the filter or VTE. Anticoagulation was not resumed following IVC filter insertion in 49 patients; consequently, the adjusted IVC filter removal rate was 69%.

Among patients who had successful removal of their filter, the median time that the IVC filter remained in place was 51 (IQR: 20–106) days. At least 10.8% (95% CI 6.4–17.7%) of patients (n = 13) experienced a complication relating to their IVC filter, including recurrent DVT (n = 4; 3.3%), IVC thrombotic occlusion (n = 3; 2.5%), filter migration (n = 3; 2.5%), recurrent PE (n = 2; 1.7%), and IVC penetration (n = 1; 0.8%). No statistical association was found between duration of filter in place for greater than 30 days and filter-related complication [*X*^2^ (1, N = 120) = 0.87, *p* > 0.05)]. Filter-related complication rates were similar among patients with guideline-congruent indications for IVC filter insertion and those with filters inserted for indications not supported by current guidelines [ *X*^2^ (1, N = 120) = 0.86, *p* > 0.05)].

Rates of filter removal were markedly reduced among patients with cancer. Fifty-four (47.5%) patients had active cancer at the time of filter insertion, and only 19 (35.2%) had their filter successfully removed. Three patients with active cancer (5.6%) experienced a filter-related complication, including filter migration (n = 2; 3.7%) and IVC thrombosis (n = 1; 1.9%). There was no significant association between the presence of active cancer and filter-related complications [ X^2^ (1, N = 120) = 2.83, *p* > 0.05)].

We categorized a subgroup of patients with poor prognosis (which we defined as death within 3 months following IVC filter insertion) and cancer-related deaths to determine if these patients may have been susceptible to worse outcomes. Out of the 18 patients that fit this criterion, only one patient had successful removal of their filter (5.6%) and 3 patients had complications (16.7%), including recurrent DVT (n = 2, 11.1%) and IVC thrombotic occlusion (n = 1, 5.6%).

## Discussion

We explored the practice patterns of IVC filter use at our institution, which is a large community-based hospital in Ontario, Canada with academic affiliations that provides care to a population of approximately 400,000 people. Our study revealed two main issues: (1) IVC filters are used in clinical settings either not supported or addressed by current major guidelines and, (2) lack of follow-up post-IVC filter insertion to ensure adequate retrieval once anticoagulation has been resumed. These issues are not unique to our institution and reflect the lack of standardization of care relating to the use of IVC filters, in general^22^.

One-third of IVC filters were inserted for indications either not addressed or supported by major clinical guidelines. While current guidelines agree that IVC filters should be considered for patients with acute VTE and contraindications to anticoagulation^3–6,23–25^, this recommendation fails to clearly define the optimal timing of IVC filter insertion among individuals with acute VTE, recognizing that the risk of recurrent VTE is highest in the initial 2–4 weeks of diagnosis^26^. The updated 2021 ACCP guidelines define acute VTE as one that is diagnosed within the preceding month; however, most other guidelines do not provide a timeframe. Moreover, patients presenting with a transient contraindication to anticoagulation following acute VTE may derive limited benefit from a filter, while risking exposure to filter-related complications^27^.

As well, the utility of IVC filters in individuals with acute PE without concurrent lower extremity DVT is not known, and similarly not supported by the updated 2021 ACCP guidelines. Nearly one third of patients in our review had PE without DVT. Other indications vary among the guidelines and remain vaguely defined, such as anticoagulant failure, massive PE with residual lower extremity DVT, etc. For example, the 2020 SIR and 2021 ACCP guidelines suggest that a filter should not be placed in patients who have recurrent VTE despite therapeutic anticoagulation, while the 2011 AHA, 2019 ESC (European Society of Cardiology), and 2020 NICE (The National Institute for Health and Care Excellence) guidelines recommend a filter in this instance^3–6,23–25^.

We identified a 10.8% complication rate associated with IVC filters in our patient cohort, which is comparable to other studies which have reported complication rates from 0 to 41%^15,28,29^. Given that the risk of complications increases with time that the filter is in situ, patients should have their filters removed as soon as possible following anticoagulation resumption^17,30^. All of the IVC filters in our study were retrievable, yet just half of them were removed. Moreover, 69.2% of patients in our study with indwelling IVC filters had resumed full-dose anticoagulant therapy. Removal rates of retrievable IVC filters vary widely between institutions, ranging from 12 to 45% in one systematic review in 2011 and under 58% in another review in 2018^14,31^. In our study, patients with active malignancy represented a significant proportion of the patients who failed to have their filter removed (31.7%) and experienced high rates of filter-related complications, suggesting that patients with advanced malignancy may derive limited benefit from IVC filters.

Our study has several limitations, including the retrospective design which is subject to confounding factors. Subjective interpretation of the guidelines may have also limited our study, as there is not a uniform definition of an acute VTE, contraindications to anticoagulation, and failure of anticoagulation, further highlighting the uncertainty surrounding current available guidelines for IVC filter use. Although complications rates differ across filter types, all participants in this study received the Cook Celect filter^32^, so we were unable to assess the effect of filter type on the complication rate. Given the retrospective nature of this study, we were also unable to access information regarding the structural integrity of the retrieved filters, but this would have provided more information on the reliability of these filters. Moreover, we only observed patterns of filter use from 2018 to 2022 to analyze the recent practice patterns in the context of current guidelines, which limited the number of filter-related events included in this study.

Our study has several strengths, including review of recent data of a moderate patient size from a large community-based hospital without a dedicated Thrombosis service, which is reflective of many other community hospitals. The results of our study have helped to improve local practice patterns relating to IVC filter use. We have developed a novel pathway whereby patients who undergo IVC filter insertion receive automatic follow-up through the Interventional Radiology department within 2–4 weeks for consideration of filter removal.

Given that the majority of clinical medicine takes place in community hospitals, it is imperative to better understand the knowledge gaps in community-based practices that have the potential to impact the care of a significant number of patients. Moreover, published data from academic centers highlight the practice variability that exists relating to the use of IVC filters in general, regardless of center of practice.

## Conclusions

Conflicting data from major guidelines likely contribute to the significant variation in use of IVC filters and may predispose towards patient harm. Given that the majority of patient care occurs in the community setting, further efforts are needed to understand patterns of IVC filter use in these hospital settings. As well, our findings further support the need for development of concrete, universally-accepted guidelines on indications for IVC filter use and improved monitoring practices post-insertion to minimize patient morbidity.

## Data Availability

The datasets generated and analyzed during the current study are available from the corresponding author on reasonable request.
